# Oxidized LDL stimulates PKM2-mediated mtROS production and phagocytosis

**DOI:** 10.1016/j.jlr.2025.100809

**Published:** 2025-04-16

**Authors:** Jue Zhang, Jackie Chang, Vaya Chen, Mirza Ahmar Beg, Wenxin Huang, Lance Vick, Yaxin Wang, Heng Zhang, Erin Yttre, Ankan Gupta, Mark Castleberry, Ziyu Zhang, Wen Dai, Jieqing Zhu, Shan Song, Moua Yang, Ashley Kaye Brown, Zhen Xu, Yan-Qing Ma, Brian C. Smith, Jacek Zielonka, James G. Traylor, Cyrine Ben Dhaou, A Wayne Orr, Weiguo Cui, Yiliang Chen

**Affiliations:** 1 Versiti Blood Research Institute, Milwaukee, WI, USA; 2 Department of Biochemistry, Medical College of Wisconsin, Milwaukee, WI, USA; 3 Department of Pediatrics, Medical College of Wisconsin, Milwaukee, WI, USA; 4 Department of Medicine, Medical College of Wisconsin, Milwaukee, WI, USA; 5 Department of Pathology, Hebei Medical University, Shijiazhuang, Hebei, China; 6 Hebei Key Laboratory of Kidney Diseases, Shijiazhuang, Hebei, China; 7 Bloodworks Northwest Research Institute, Seattle, WA, USA; 8 Division of Hematology and Oncology, Department of Medicine, University of Washington School of Medicine, Seattle, WA, USA; 9 Department of Microbiology and Immunology, Medical College of Wisconsin, Milwaukee, WI, USA; 10 Department of Pathology, Northwestern University, Chicago, IL, USA; 11 Department of Biophysics, Medical College of Wisconsin, Milwaukee, WI, USA; 12 Department of Pathology and Translational Pathology, LSU Health Shreveport, LA, USA; 13 Medical College of Wisconsin, Cardiovascular Center, Milwaukee, WI, USA

**Keywords:** CD36, macrophages, phagocytosis, glycolysis, PKM2, mitochondria, reactive oxygen species, atherosclerosis

## Abstract

Oxidized low-density lipoprotein (oxLDL) promotes proatherogenic phenotypes in macrophages, accelerating the progression of atherosclerosis. Our previous studies demonstrated that oxLDL binds to its receptor CD36, stimulating mitochondrial reactive oxygen species (mtROS), which are critical in atherosclerosis development. However, the mechanisms underlying mtROS induction and their effects on macrophage cellular functions remain poorly understood. Macrophages rely on phagocytosis to clear pathogens, apoptotic cells, or other particles, a process critical for tissue homeostasis. Dysregulated or excessive particle ingestion, a key step in phagocytosis, can lead to lipid overloading and foam cell formation, a hallmark of atherosclerosis. In this study, we showed that macrophages pretreated with oxLDL exhibit increased particle ingestion, a phagocytic response significantly attenuated in *Cd36*-null macrophages. Further investigations revealed that oxLDL-induced phagocytosis depends on mtROS, as their suppression inhibited the process. In vivo, atherosclerosis-prone *Apoe*-null mice on a high-fat diet exhibited increased mtROS levels and enhanced phagocytic activity in aortic foamy macrophages compared to those from chow diet–fed mice, supporting a role of mtROS in promoting lesional macrophage phagocytosis. Mechanistically, we identified a novel signaling pathway whereby oxLDL/CD36 interaction induces the translocation of the cytosolic enzyme pyruvate kinase muscle 2 (PKM2) to mitochondria. Disruption of PKM2 mitochondrial translocation using siRNA knockdown or a specific chemical inhibitor reduced mtROS production and attenuated oxLDL-induced phagocytosis. In conclusion, our findings reveal a novel oxLDL-CD36-PKM2 signaling axis that drives mtROS production and phagocytosis in atherogenic macrophages.

## Graphical abstract

**Figure:**
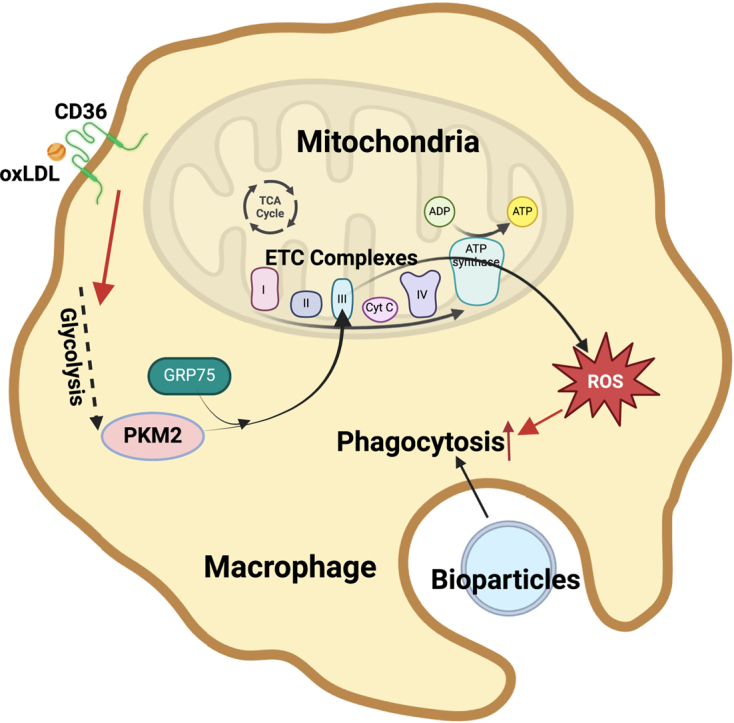

Macrophages play a central role in tissue homeostasis by eliminating pathogens, apoptotic cells (AC), or other large particles through phagocytosis. Phagocytosis is a multistep cellular process that involves the recognition, binding, ingestion, and digestion of particles. All steps are tightly regulated and coordinated to ensure proper handling of the internalized particles (1). Since many of those particles contain various lipid molecules, excessive or dysregulated phagocytosis imposes a substantial metabolic burden on macrophages, leading to intracellular lipid accumulation and foam cell formation—key features in the initiation of atherosclerosis (2, 3).

Retained lipoproteins in the arterial subendothelial space undergo modifications, generating atherogenic particles such as oxidized LDL (oxLDL). OxLDL binds to macrophage surface receptors, including CD36 (4). Subsequently, CD36 mediates endocytosis of oxLDL, promoting lipid accumulation and foam cell formation (5, 6). Our previous work demonstrated that oxLDL/CD36 signaling reprograms macrophage metabolism, shifting from mitochondrial respiration to glycolysis, accompanied by mitochondrial alterations, including increased fission and elevated mitochondrial reactive oxygen species (mtROS) production (7). In a diet-induced atherosclerosis model (*Apoe*-null mice), we observed persistently elevated mtROS levels in circulating monocytes in a CD36-dependent manner during the early stages of atherogenesis (7). Mitochondrial dysfunction has been closely linked to atherosclerosis in both animal models and human patients (8). Furthermore, suppression of mtROS through a transgenic approach (mitochondrial-specific human catalase overexpression, MCAT) protected mice from early atherogenesis, highlighting a contributory role for mtROS (9).

While previous studies, including ours, have connected mtROS to proinflammatory signaling pathways such as NF-κB activation and cytokine production (7, 9), two critical questions remain unresolved: (1) How does oxLDL-induced mtROS influence macrophage cellular functions such as phagocytosis? (2) What is the molecular mechanism underlying mtROS induction by oxLDL? In this study, we aimed to address both questions.

## Materials and methods

### Experimental animals

All mice used in this study were on the C57BL/6 background. WT mice were from Charles River. *Apoe*-null mice and MCAT transgenic mice were purchased from the Jackson Laboratory. *Cd36* null, *Apoe*/*Cd36* double-null were generated as previously described (10). MCAT mice were crossed with *Apoe*-null mice to generate *Apoe*-null/MCAT for the in vivo phagocytosis assays. All mice were kept in a 12-h dark/light cycle and fed standard chow ad libitum unless indicated otherwise. Animals of both sexes were used, but since we have not observed sex differences in macrophage CD36-PKM2 signaling axis, data were analyzed together. For high-fat diet (HFD) challenge experiments, adult mice between 16 and 20 weeks of age were randomly divided into two groups: one was continued on a standard chow diet and the other was placed on an atherogenic HFD (Harlen Teklad, #TD.88137) for 6 weeks before assays. The HFD contains 0.2% cholesterol and 21% total fat by weight with 42% kcal from fat. It is widely used for inducing atherosclerosis in mice. All procedures involving live animals were approved by the Institutional Animal Care and Use Committee at the Medical College of Wisconsin.

### Human artery samples

Human aorta and coronary artery samples were collected by Dr Wayne Orr’s lab (Louisiana State University), embedded in paraffin, and atherosclerotic plaques were scored by a pathologist based on the Stary Classification System (11). The samples were subjected to deparaffinization, antigen retrieval with 20 μg/ml proteinase K (Abcam, #ab64220) 5-min incubation at room temperature, and stained using anti-4-HNE (Antibodies-online.com, #ABIN873270) and anti-CD68 (Abcam, #ab955). The secondary antibody Alexa Fluor 647–conjugated antibody (Abcam, #ab150075) targeted the 4-HNE antibody, and Alexa Fluor 405–conjugated antibody (Abcam, #ab175660) targeted the CD68 antibody. This study, conducted exclusively with postmortem human tissue (STUDY00001712), has been classified as nonhuman research and approved by the Institutional Review Board at LSU Health Shreveport.

### Preparation of LDL, oxLDL, and HDL

Human LDLs (#360-10) were purchased from Lee BioSolutions (MO). LDLs were diluted and oxidized as described here. Briefly, oxidation of LDL (0.5 mg/ml) by Cu^2+^ was performed by dialysis versus 5 μM CuSO_4_ in PBS for 6 h at 37°C. Oxidation was terminated by adding EDTA (0.54 mM). Then, the oxLDL was washed twice with PBS to remove Cu^2+^. LDL oxidation was confirmed by thiobarbituric acid reactive substances assay using a kit (Abcam, #ab118970). The oxLDL stock solution (0.5 mg/ml) was put in a 15 ml tube with argon gas flushed above and parafilm wrapped around the cap before the tube was stored in a 4°C fridge until usage. Human HDLs (#361-25) were also purchased from Lee BioSolutions.

### Isolation and in vitro culture of peritoneal macrophages

Mice were injected with 2 ml 4% thioglycolate in sterile saline intraperitoneally and 4 days later were sacrificed with CO_2_. Peritoneal cavities were flushed, and macrophages were then suspended in 10 ml PBS prewarmed to 37°C, counted, and centrifuged at 250*g* for 5 min. Cells were resuspended in the culture media and seeded into culture dishes. Cells were cultured in RPMI media supplemented with 10% FBS, 100 U/ml penicillin, and 100 μg/ml streptomycin at 37°C in a humidified incubator with 5% CO_2_. Cells were maintained in full media for at least 48 h before any treatment. All subsequent treatments were conducted in RPMI media in the presence of 10% FBS.

### Isolation and *in vitro* culture of human monocyte-derived macrophages

Monocytes were isolated from human buffy coats by Ficoll density centrifugation and allowed to differentiate over 7 days in vitro to macrophages as described (6). Human monocyte-derived macrophages (HMDMs) were cultured in X-ViVo 15 hematopoietic media supplemented with 5% human serum, 100 U/ml penicillin, and 100 μg/ml streptomycin at 37°C in a humidified incubator with 5% CO_2_. All following treatments were conducted in X-ViVo 15 media in the presence of 5% human serum.

### In vitro phagocytosis assays

Macrophages were seeded into 6-well plates at 0.25 × 10^6^ cells/well and pretreated with or without oxLDL for 24 h. Cells were then incubated with 20 μg/ml pHrodo-conjugated *E. coli* bioparticles (Invitrogen, #P35366) for 30 min. Cells were washed with PBS twice to remove free bioparticles, lifted by scraping, and suspended for flow cytometry analysis using the FITC channel. The pHrodo fluorescence signal intensity was quantified as an indicator of phagocytosis activity. In a second phagocytosis protocol, cells were incubated with 10 um green fluorescence beads (Thermo Fisher Scientific, #G0100) diluted in RPMI1640 medium in the incubator for 16 h. The cells were washed with PBS twice to remove free beads, lifted by scraping, and suspended for flow cytometry analysis using the FITC channel. The GFP signal intensity was quantified as an indicator of phagocytosis activity. In a third phagocytosis protocol, cells were incubated with PKH67 (Sigma, #MINI67)-labeled AC for 15 min. Unbound AC were washed away by PBS for three times. Then macrophages were lifted by scraping and suspended for flow cytometry analysis using the FITC channel. The PKH67 fluorescence signal intensity was quantified as an indicator of phagocytosis activity.

### Flow cytometry assays

Each cell suspension was prepared in 200 μl flow buffer (PBS with 5% FBS and 0.1% sodium azide). All flow cytometry experiments were performed with a BD LSR II instrument using FACSDiva software with optimal compensation and gain settings determined for each experiment based on unstained and single color-stained samples. Live cells were gated based on cell forward and side scatter signals. Doublets were excluded based on forward scatter (FSC)-A versus FSC-H plots. FlowJo software version 10.8.1 (Tree Star, OR) was used to analyze the data.

### Measurement of macrophage mtROS

Cells were seeded into 6-well plates at 0.25 × 10^6^ cells/well. After treatment, cells were incubated with 5 μM MitoNeoD for 15 min. Cells were washed with PBS twice, lifted by scraping, and suspended for flow cytometry analysis using the phycoerythrin (PE) channel.

### Ex vivo aortic F4/80^+^/Trem2^+^ macrophage mtROS assays and phagocytosis assays

Mice were sacrificed after 6 weeks of chow/HFD. The whole aortas were removed, digested in gentle MACSTM C Tubes (Miltenyi Biotec) with an enzyme mixture (Liberase, 0.77 mg/ml; hyaluronidase, 0.3 mg/ml; deoxyribonuclease, 0.3 mg/ml; BSA, 1 mg/ml; CaCl_2_ 1.5 uM, in PBS) by a gentle MACSTM Octo Dissociator with Heaters (Miltenyi Biotec) at 37°C for 45 min. Cell suspension was then filtered by a 40 μm cell strainer to remove the large debris. For mtROS assays, the filtered single-cell suspension was incubated with 5 μM MitoNeoD in RPMI1640 with 10% FBS at 37°C for 15 min, followed by immunostaining with Pacific Blue–conjugated F4/80.

Antibody (BioLegend, #123123) and APC-conjugated Trem2 antibody (R&D Systems, #FAB17291A) in flow buffer at room temperature for 15 min in the dark. Cells were then washed once with flow buffer and resuspended in 200 μl flow buffer for flow cytometry analysis. For phagocytosis assays, the filtered single-cell suspension was incubated with 20 μg/ml pHrodo-conjugated *E. coli* bioparticles in RPMI1640 with 10% FBS at 37°C for 30 min, followed by immunostaining with PE/Cy5-conjugated F4/80 antibody (BioLegend, #123111) and PE-conjugated Trem2 antibody (R&D Systems, #FAB17291P) in flow buffer at room temperature for 15 min in the dark. Cells were then washed once with flow buffer and resuspended in 200 μl flow buffer for flow cytometry analysis.

### In vivo aortic F4/80^+^/Trem2^+^ macrophage phagocytosis assays

At 6 weeks on chow/HFD, each mouse was injected with 50 μg pHrodo-conjugated *E. coli* bioparticles through the tail vein. Mice were sacrificed 3 h later, and the aortas were processed as described in the ex vivo phagocytosis assay. The pHrodo bioparticle incubation step was skipped.

### PKM2 immunoprecipitation and mass spectrometry for proteomics analysis

WT murine peritoneal macrophages were treated with 20 μg/ml oxLDL for 3 h. Cells were lysed, and 1 mg of protein was incubated with 2 μg anti-PKM2 IgG (Cell signaling, #4053) at 4°C overnight. Then, 25 μl of A/G agarose beads were added and incubated at 4°C overnight. The beads were pelleted at 10,000*g* for 1 min at room temperature, washed three times with lysis buffer, boiled in loading buffer, and the supernatant was loaded to SDS-PAGE gel for electrophoresis. The gel was stained by Coomassie Protein Stain (Abcam) and the gel lanes were excised, including three from control PKM2 immunoprecipitation (IP) samples and three from oxLDL-treated PKM2 IP samples, a blank gel (negative control #1) and a control IgG IP sample (negative control #2). The excised gels were subjected to mass spectrometry proteomics analysis by the Harvard Medical School Taplin Mass Spectrometry Facility. Proteins identified in the two negative controls were considered false positives and excluded from further analysis.

### Mitochondria/cytosol fractionation

Macrophages were subjected to mitochondria/cytosol fractionation using a commercial Mitochondria Isolation kit (Thermo Fisher Scientific) following the manufacturer’s instruction.

### Transfection of siRNA

The siRNAs against *HSPA9* (encoding GRP75) and *PKM* (encoding PKM2) were obtained from Integrated DNA Technologies and mixed with INTERFERin® transfection reagent (Polyplus) for 10 min at room temperature temperature before transfection. After 10 days differentiation with ∼70% confluency, HMDMs were transfected with siRNA duplexes at 50 nM concentration in serum-free medium for 24 h and then replaced with fresh full medium for another 24 h. The scrambled siRNA (Qiagen) was used as the negative control.

#### Human HSPA9/GRP75 siRNA duplex sequence:

5′- rGrUrA rUrCrA rGrCrA rUrGrU rGrCrA rArArU rCrUrU rGrUT T -3′

5′- rArArA rCrArA rGrArU rUrUrG rCrArC rArUrG rCrUrG rArUrA rCrUrG -3′

#### Human PKM siRNA duplex sequence:

5′ - rGrArU rUrArU rCrArG rCrArA rArArU rCrGrA rGrArA rUrCA T -3′

5′ - rArUrG rArUrU rCrUrC rGrArU rUrUrU rGrCrU rGrArU rArArU rCrUrU -3′

### IP and immunoblot analysis

Cells were washed with PBS and lysed with ice-cold CelLytic M Cell Lysis Reagent containing protease inhibitor cocktail (Roche) and phosphatase inhibitors (Sigma). For IP assays, the lysates were precleared with A/G agarose beads (Thermo Fisher Scientific) at 4°C for 1 h. The supernatants containing the same amount of protein (∼1 mg) were incubated with 10 μl primary antibody overnight at 4°C; 25 μl of A/G agarose beads was then added and incubated overnight at 4°C. The beads were washed, boiled in loading buffer, and the supernatant was loaded to SDS-PAGE gel. For immunoblotting, protein concentrations were determined by the NanoDrop One/OneC spectrophotometer (Thermo Fisher Scientific) and equal amounts of proteins were loaded in each lane. The signals were detected with chemiluminescent substrate (Thermo Fisher Scientific) and quantified by ImageJ software. The primary antibodies used include: anti-PKM2 (Cell Signaling, #4053), anti-ATP5A (Santa Cruz, #sc-136178), anti-β-actin (Sigma, #A5316), anti-Tom20 (Abcam, #ab56783), anti-GRP75 (Cell Signaling, #3593), anti-UQCRC1 (Invitrogen, #459140), and anti-Cytochrome c (Cell Signaling, #4272).

### Immunofluorescence assay

Macrophages were seeded on uncoated glass coverslips and fixed in 4% paraformaldehyde for 15 min, permeabilized in 0.2% Triton X-100 for 10 min, and blocked with 3% BSA for 1 h. The cells were incubated with primary antibodies overnight at 4°C and then fluorescence-conjugated secondary antibodies for 3 h at room temperature in the dark. Cells were then washed and counterstained with 4′,6-diamidino-2-phenylindole Vectashield mounting medium and imaged by laser confocal fluorescence microscopy (Olympus). For colocalization quantification based on two-color immunostaining, images were analyzed using the software Nikon NIS-Elements. Signals from each channel were first quantified separately and the total area from each color was determined. Then, the area with both signals present was quantified as the colocalization area. Finally, the colocalization index was expressed as the percentage of colocalization area among total mitochondria area in each cell.

### In situ proximity ligation assay

Macrophages were fixed and incubated with rabbit anti-PKM2 (Cell Signaling, Cat#4053) and mouse anti-UQCRC1 monoclonal antibodies (Invitrogen, Cat#459140). Cells were then washed and incubated with species-specific secondary antibodies (Olink) conjugated to unique DNA strands. Negative control slides were only incubated with secondary antibodies. The oligonucleotides and a ligase were added to form a circular template which was then amplified and detected using complementary fluorescently labeled probes. Red fluorescent dots representing protein-protein interaction were visualized by laser confocal fluorescence microscope.

### Single-cell RNA-seq reanalysis

Single-cell RNA sequencing (ScRNA-seq) data from Gene Expression Omnibus Database (GSE97310) were reanalyzed using the Seurat package.

### Statistics

Data are presented as means ± SEM. In all the figure legends, n values represent biological replicates. All statistical analysis was done using GraphPad Prism 9 software. D’Agostino-Pearson omnibus normality test was performed to confirm that data are normally distributed followed by the Student’s *t* test (two-tailed, unpaired, and equal variance) for comparisons between two groups. For data comparison involving more than two groups, ANOVA was used followed by Dunnett’s or Tukey’s multiple comparisons test. Statistical values, including number of replicates (n), are noted in the figure legends. In all figures, “ns” means not significant. ∗*P* < 0.05, ∗∗*P* < 0.01, ∗∗∗*P* < 0.001, and ∗∗∗∗*P* < 0.0001. For in vitro studies n = number of biological repeats while for the in vivo studies, n = number of animals.

## Results

### OxLDL stimulates phagocytosis in macrophages

**Fig. 1:**
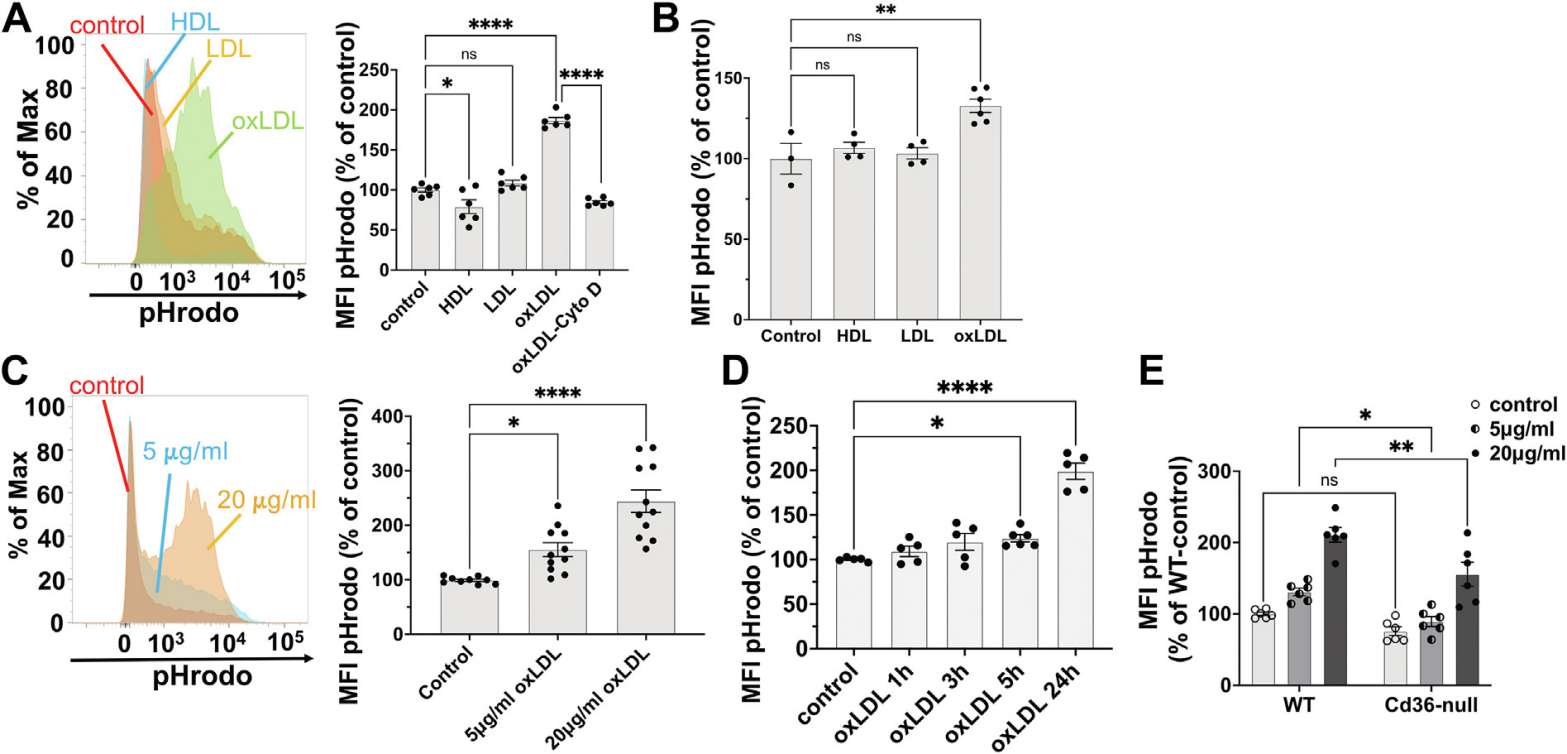
OxLDL stimulates phagocytosis in macrophages. A: Examples of histograms of pHrodo fluorescence in WT peritoneal macrophages pretreated with 20 μg/ml HDL, LDL, or oxLDL for 24 h, followed by pHrodo-conjugated *E. coli* coincubation and flow cytometry analysis. For oxLDL plus cytochalasin D condition, 5 μM cytochalasin D was added 30 min before the phagocytosis assay. The pHrodo MFI was quantified and shown in the bar graph; n = 3 per group. B: HMDMs were pretreated with 50 μg/ml HDL, LDL, or oxLDL for 24 h before the phagocytosis assay. The pHrodo MFI was quantified and shown in the bar graph; n = 3 per group. C: Examples of histograms of pHrodo fluorescence in WT peritoneal macrophages pretreated with 5 or 20 μg/ml of oxLDL for 24 h before phagocytosis assay. MFIs are shown in the bar graph; n = 4 per group. D: WT peritoneal macrophages were pretreated with 20 μg/ml oxLDL for the indicated periods before the phagocytosis assay. MFIs are shown in the bar graph; n = 3 per group. E: WT or *Cd36*-null peritoneal macrophages were pretreated with 5 or 20 μg/ml oxLDL for 24 h before the phagocytosis assay. The pHrodo MFI was quantified and shown in the bar graph; n = 3 per group. Max, maximum fluorescence intensity; ns, not significant; ∗*P* < 0.05; ∗∗*P* < 0.01; and ∗∗∗∗*P* < 0.0001. HMDM, human monocyte-derived macrophage; MFI, mean fluorescence intensity; OxLDL, oxidized low-density lipoprotein.

We pretreated WT murine peritoneal macrophages with 20 μg/ml oxLDL for 24 h, followed by a 30-min incubation with pHrodo-conjugated *E. coli* bioparticles. The pHrodo fluorescence, which intensifies in acidic phagolysosomes, was measured using flow cytometry to quantify the ingested bioparticles, reflecting phagocytic activity (12). OxLDL treatment increased the activity by 87%, an effect inhibited by cytochalasin D. Nevertheless, non-oxLDL or HDL pretreatment did not increase phagocytosis (Fig. 1A). HMDMs also exhibited enhanced phagocytosis upon oxLDL treatment, with a 33% increase in activity (Fig. 1B).

OxLDL stimulation was dose-dependent (Fig. 1C) and time-dependent (Fig. 1D), with significant effects observed as early as 5 h posttreatment (24% increase). This effect extended beyond *E. coli* bioparticles, as oxLDL also enhanced the phagocytosis of polystyrene beads (supplemental Fig. S1A) and AC (supplemental Fig. S1B).

The scavenger receptor CD36 is a known oxLDL receptor in macrophages (4). Macrophages lacking CD36 exhibited attenuated phagocytosis in response to oxLDL treatment (Fig. 1C, E), suggesting oxLDL stimulates phagocytosis through CD36.

### OxLDL-stimulated phagocytosis is dependent on mtROS

**Fig. 2:**
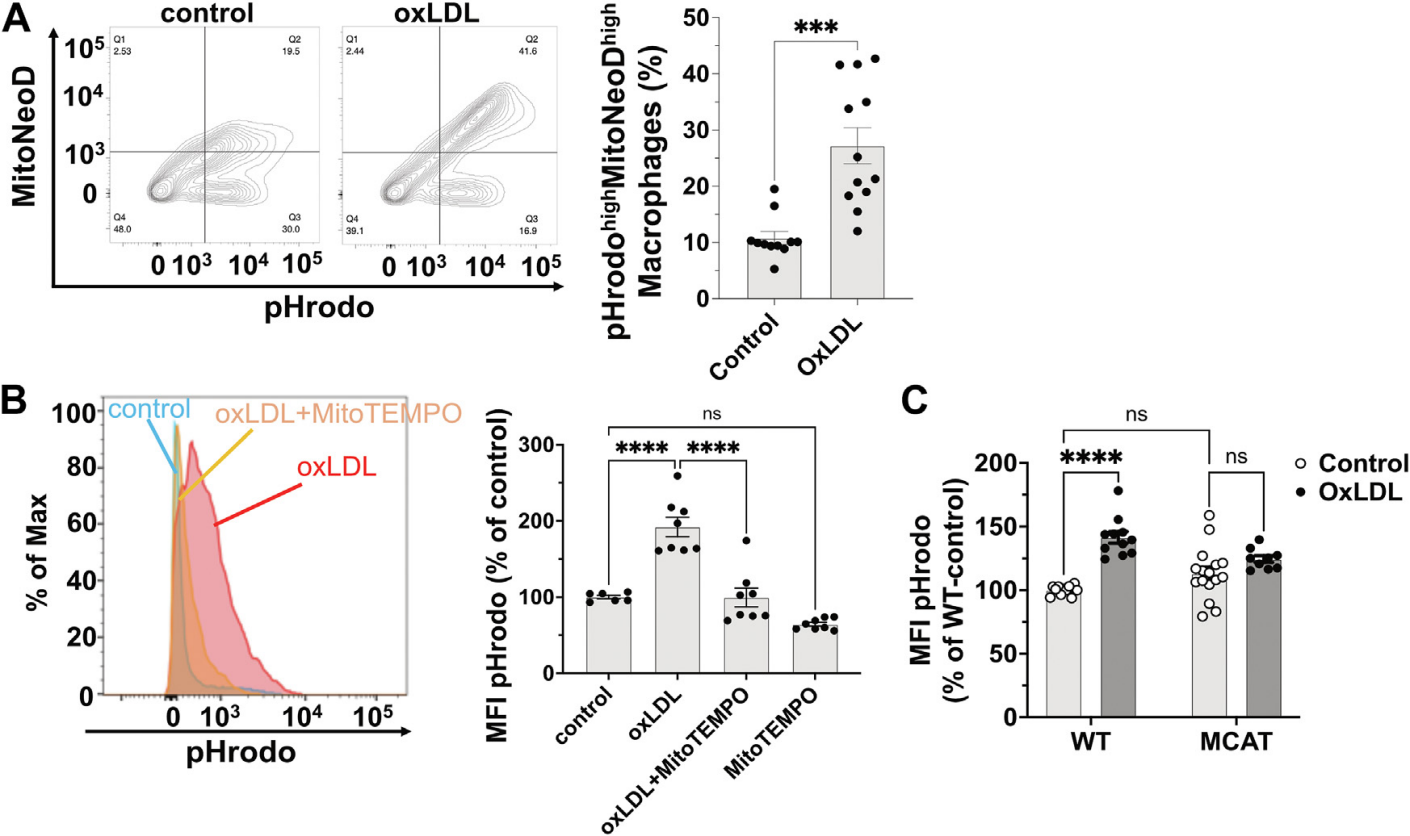
OxLDL-stimulated phagocytosis is dependent on mitochondrial ROS. A: Examples of contour plots showing MitoNeoD and pHrodo signals from WT peritoneal macrophages pre-treated with 20 μg/ml oxLDL for 24 h. The percentage of MitoNeoD/pHrodo double positive populations was quantified and shown in the bar graph; n = 3–5 per group. B: Examples of histograms of pHrodo fluorescence in WT peritoneal macrophages pre-treated with 20 μg/ml oxLDL alone or in combination with 1 mM MitoTEMPO. MitoTEMPO was added 1 h before the assay. The pHrodo MFI was quantified and shown in the bar graph; n = 3 per group. C: WT or MCAT peritoneal macrophages were pretreated with 20 μg/ml oxLDL for 5 h before the phagocytosis assay. The pHrodo MFI was quantified and shown in the bar graph; n = 4–5 per group. Max, maximum fluorescence intensity; ns, not significant; ∗∗∗*P* < 0.001; and ∗∗∗∗*P* < 0.0001. MFI, mean fluorescence intensity; MCAT, mitochondrial-specific human catalase overexpression; OxLDL, oxidized low-density lipoprotein; ROS, reactive oxygen specie.

We previously reported that oxLDL increased mtROS levels via CD36 in macrophages (7). To determine whether mtROS contribute to oxLDL-induced phagocytosis, we costained macrophages with MitoNeoD, a specific mitochondria-targeted fluorescent redox probe (7, 13), and pHrodo-conjugated *E. coli* bioparticles. OxLDL pretreatment significantly increased a macrophage subpopulation exhibiting high mtROS and pHrodo signals (Fig. 2A), suggesting a positive correlation between mtROS levels and phagocytosis activity. The mtROS scavenger MitoTEMPO blocked oxLDL-induced phagocytosis (Fig. 2B), supporting the role of mtROS in this process.

To validate these findings, we utilized peritoneal macrophages from MCAT mice, which overexpress mitochondrial catalase to suppress mtROS (9). MCAT macrophages were resistant to oxLDL-stimulated phagocytosis, while basal phagocytic activity remained unaffected (Fig. 2C).

### Aortic foamy macrophages exhibit increased mtROS-Dependent phagocytosis activity

**Fig. 3:**
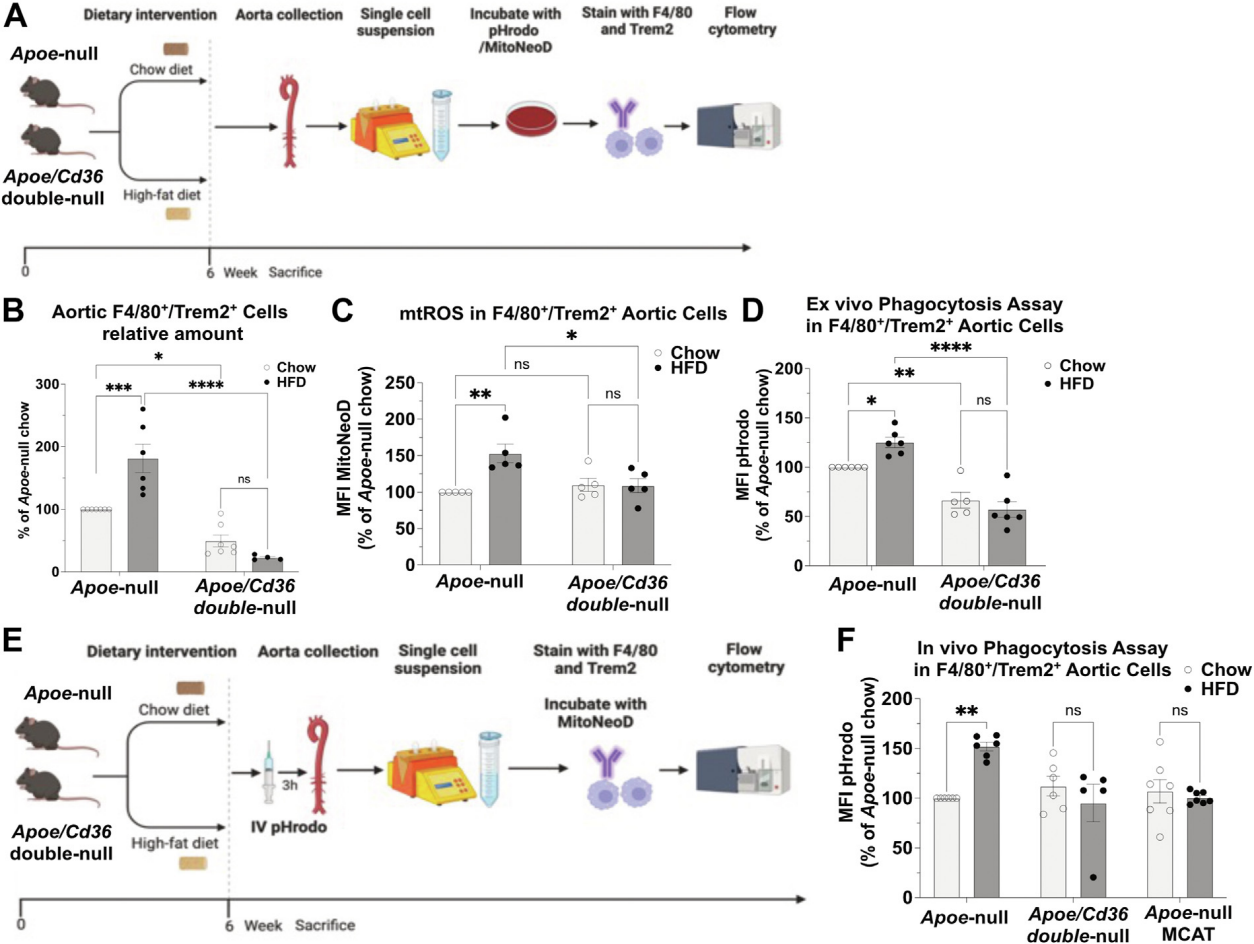
Aortic foamy macrophages exhibit increased mtROS-dependent phagocytosis activity. A: A diagram of the ex vivo mtROS and phagocytosis assay. B: Total amounts of aortic F4/80^+^/Trem2^+^ macrophages were quantified and shown in the bar graph; n = 4–7 individual mice per group. C: The MitoNeoD MFI was quantified in aortic F4/80^+^/Trem2^+^ macrophages and shown in the bar graph; n = 5 individual mice per group. D: The pHrodo MFI was quantified in aortic F4/80^+^/Trem2^+^ macrophages and shown in the bar graph; n = 5–6 individual mice per group. E: A diagram of the in vivo phagocytosis assay. F: The pHrodo MFI was quantified in aortic F4/80^+^/Trem2^+^ macrophages and shown in the bar graph; n = 5–7 individual mice per group. ns, not significant; ∗*P* < 0.05; ∗∗*P* < 0.01; ∗∗∗*P* < 0.001; and ∗∗∗*P* < 0.0001. MFI, mean fluorescence intensity; mtROS, mitochondrial reactive oxygen species.

OxLDL promotes lipid-laden foam cell formation and facilitates the initiation and progression of atherosclerosis (5, 6, 14). In human atherosclerotic lesions, oxidative stress marker 4-HNE (15) colocalized with the macrophage marker CD68 in regions adjacent to the lumen (supplemental Fig. S2A), suggesting an association between lesional macrophages and reactive oxygen species (ROS). In *Apoe*-null mice fed with HFD, we previously reported sustained mtROS elevation in circulating Ly6C^high^ proinflammatory monocytes during atherogenesis (7). These monocytes infiltrate lesion sites and differentiate into plaque macrophages (16, 17). To investigate the role of mtROS on phagocytosis in aortic lesional macrophages, we conducted ex vivo analysis using aortic single-cell suspensions from *Apoe*-null and *Apoe*/*Cd36* double-null mice fed chow or HFD for six weeks. Aortic cells were incubated with anti-F4/80 (macrophage marker), anti-Trem2 (aortic foamy cell marker) (18), MitoNeoD, and *E. coli*-pHrodo (Fig. 3A). Clumped cells were excluded for analysis using the FSC-A versus FSC-H gating strategy and F4/80^+^/Trem2^+^ cells were selected as foamy macrophages for analysis (supplemental Fig. S2B).

HFD increased aortic F4/80^+^ macrophages (supplemental Fig. S2C) and F4/80^+^/Trem2^+^ foamy macrophages by 81% in *Apoe*-null mice (Fig. 3B). This increase was attenuated in *Apoe*/*Cd36* double-null mice under both chow and HFD conditions, consistent with prior findings showing protection from chronic inflammation and atherosclerosis in these mice (10). Notably, mtROS levels in F4/80^+^/Trem2^+^ foamy macrophages were elevated by 53% in *Apoe*-null mice on HFD but not in *Apoe*/*Cd36* double-null mice (Fig. 3C), indicating that CD36 is required for mtROS induction. Phagocytosis activity was also increased by 25% in F4/80^+^/Trem2^+^ foamy macrophages from *Apoe*-null mice on HFD, but not in *Apoe*/*Cd36* double-null mice (Fig. 3D).

In vivo phagocytosis assays (Fig. 3E) corroborated these findings. *E. coli*-pHrodo injection followed by aortic analysis revealed a 52% increase in phagocytosis activity in F4/80^+^/Trem2^+^ foamy macrophages from *Apoe*-null mice on HFD, while no such increase was observed in *Apoe*/*Cd36* double-null or *Apoe*-null/MCAT mice (Fig. 3F). This effect was specific to aortic macrophages, with no changes detected in macrophages from adipose tissue, liver (supplemental Fig. S2D), or peripheral blood monocytes (pHrodo signals not detected in all conditions). These results indicate that CD36-dependent mtROS induction drives increased phagocytosis in aortic F4/80^+^/Trem2^+^ foamy macrophages during diet-induced atherosclerosis in mice.

### *PKM* expression is correlated with mtROS induction in plaque-forming immune cells

To investigate the molecular mechanism underlying oxLDL-induced mtROS production, we hypothesize that a second messenger downstream of the oxLDL/CD36 complex transmits signals from the cytoplasm to mitochondria, inducing mtROS. Given our previous findings that oxLDL induces a metabolic switch from oxidative phosphorylation to glycolysis (7), we tested the role of this switch in mtROS induction. Treatment with the glycolysis inhibitor 2-deoxy-D-glucose (19) significantly blocked oxLDL-induced mtROS production and phagocytosis (supplemental Fig. S3A), suggesting that glycolytic enzyme activation may be involved.

**Fig. 4:**
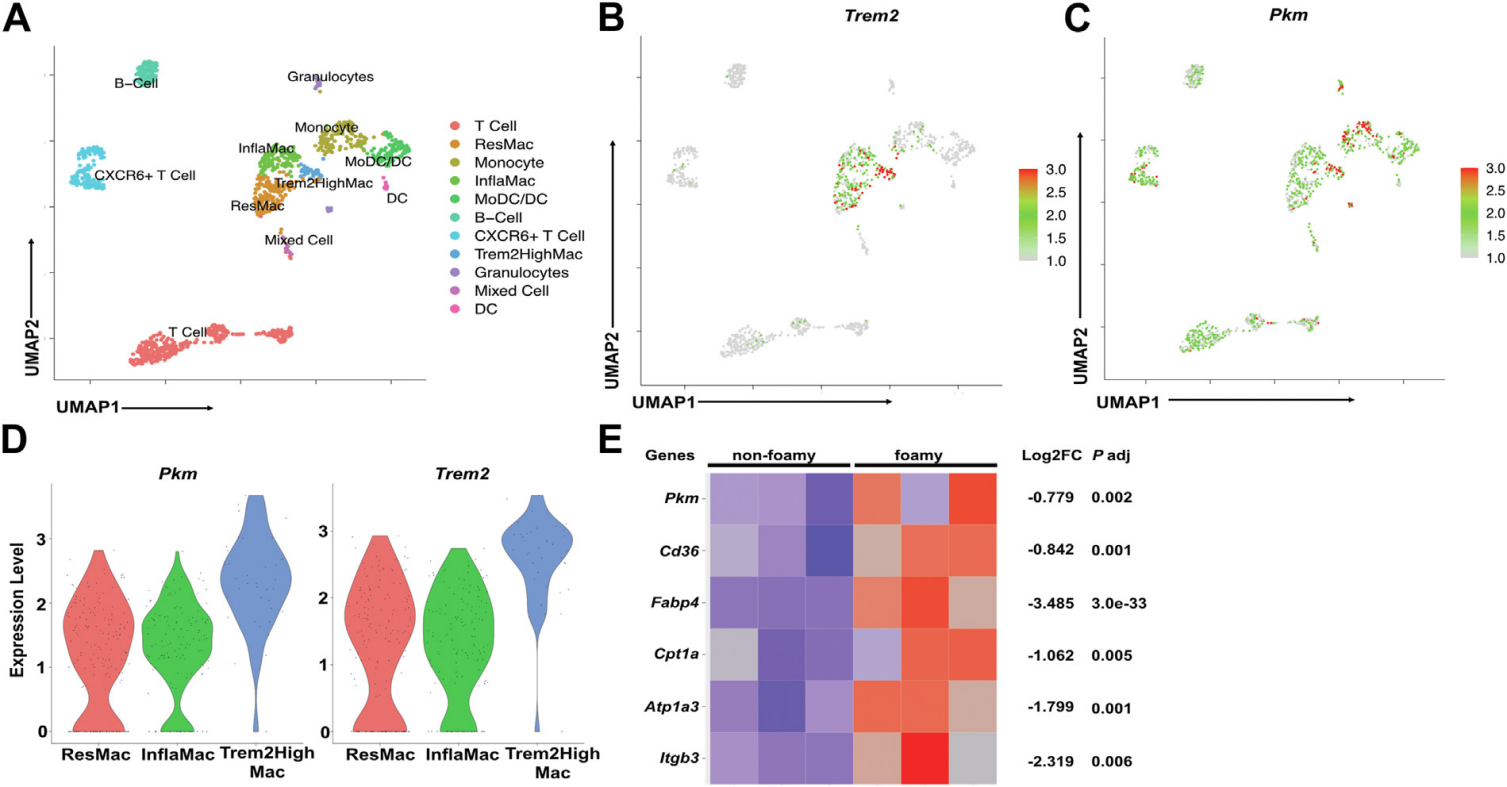
*PKM* expression is correlated with mtROS induction in plaque-forming immune cells. A–D: Mouse scRNA-seq data were reanalyzed from a published dataset (GSE97310). UMAP representation of 11 aortic CD45+ immune cell clusters is shown in (A). *Trem2* gene expression pattern (B) and *Pkm* gene expression pattern (C) are shown in the UMAP. (D) Violin plots show the *Pkm* and *Trem2* expression distribution among aortic macrophage subpopulations. ResMac, resident macrophage; InflaMac, inflammatory macrophage. E: Bulk RNA-seq data were reanalyzed from a published data set (GSE116239). A heat map is shown of differential expression of relevant genes comparing between foamy and nonfoamy macrophages from mice aorta. mtROS, mitochondrial reactive oxygen species; PMK2, pyruvate kinase muscle 2; UMAP, uniform manifold approximation and projection.

We next analyzed whether the expression pattern of glycolytic enzymes correlates with mtROS levels in aortic immune cells. Reanalysis of published scRNA-seq data (18) from CD45^+^ immune cells in mouse aortas during atherogenesis identified 11 immune cell clusters using uniform manifold approximation and projection (Fig. 4A). Trem2^high^ macrophages were identified as a distinct cluster based on previous studies (18, 20) and shown in the uniform manifold approximation and projections (Fig. 4A, B). Notably, *Pkm*, the gene encoding the glycolytic enzyme pyruvate kinase, was highly expressed in both Ly6C^high^ proinflammatory monocytes and Trem2^high^ macrophages (Fig. 4C, D). This correlated with elevated mtROS levels in Ly6C^high^ monocytes (previously reported) and F4/80^+^/Trem2^+^ foamy macrophages (Fig. 3C). It also correlated with *Prdx1* expression pattern (supplemental Fig. S3B), consistent with an antioxidant response to ROS (21) or oxLDL (7). In contrast, other genes encoding glycolytic rate-limiting enzymes (e.g., *Hk1*, *Pfkl*) showed no such correlation (supplemental Fig. S3C).

Further, *Pkm* expression was predominantly observed in immune cells from HFD-fed mice (Fig. 4C and supplemental Fig. S3D), suggesting its upregulation during atherogenesis. Reanalysis of bulk RNA sequencing data from foamy versus nonfoamy macrophages (20) confirmed elevated *Pkm* expression in lipid-laden foamy macrophages, alongside *Cd36* and its signaling partners (i.e. *Atp1a3* (6) and *Itgb3* (22)) and fatty acid trafficking partners (i.e. *Fabp4* and *Cpt1a* (7)) (Fig. 4E). These findings suggest a potential role for *PKM* gene expression in mtROS induction in lipid-enriched plaque-forming immune cells during atherogenesis.

### OxLDL stimulates PKM2 mitochondrial translocation to induce mtROS

**Fig. 5:**
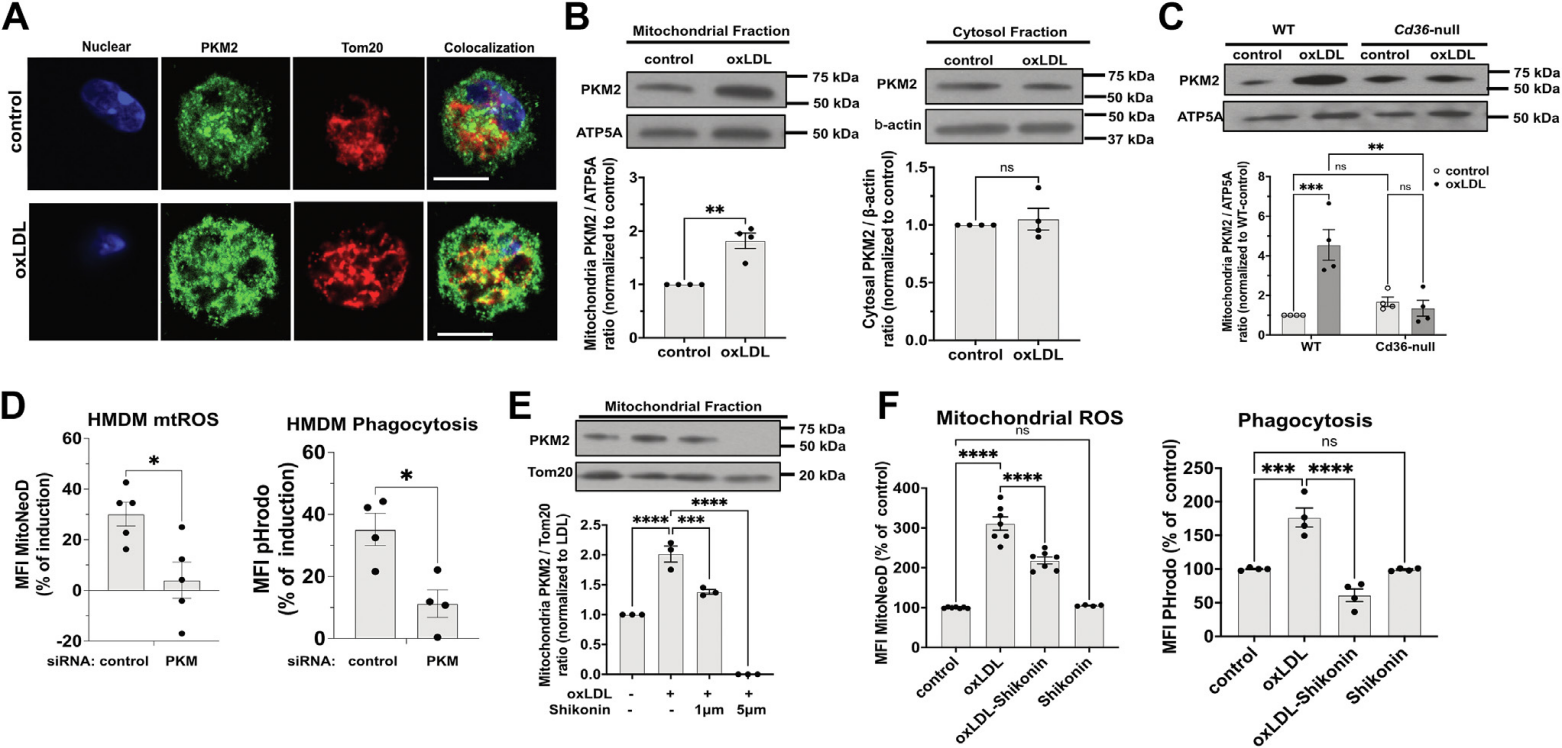
OxLDL stimulates PKM2 mitochondrial translocation to induce mtROS. A: Representative confocal images (n = 6–10) of macrophages immunostained for PKM2 (green) and Tom20 (red). Nuclei were stained by DAPI (blue). Scale bar: 5 μm. B: HMDMs treated with 50 μg/ml LDL (control) or oxLDL for 3 h were lysed and subjected to cell fractionation into mitochondrial and cytosol fractions. PKM2 and ATP5A (mitochondria fraction loading control) blot images from mitochondrial fractions were shown on the left. PKM2 and β-actin (cytosol fraction loading control) blot images from cytosol fractions are shown on the right. Images were quantified, normalized to each loading control, and expressed as fold change of control. n = 4 per group. C: WT or *Cd36*-null peritoneal macrophages were treated with 20 μg/ml LDL (control) or oxLDL for 3 h and then processed as in B. Mitochondrial fractions were immunoblotted for PKM2 and ATP5A and blot images are shown. Images were quantified, normalized, and expressed as fold change of control. n = 4 per group. D: HMDMs transfected with control or *PKM* siRNA were treated with 50 μg/ml oxLDL for 24 h before being subjected to mtROS assay (left panel) or phagocytosis assay (right panel). MFI was quantified and shown as the percent of induction by oxLDL in the bar graph; n = 4–6 per group. E: WT macrophages treated with 20 μg/ml oxLDL for 3 h or pretreated with 1 or 5 μM shikonin for 1 h before addition of oxLDL, incubating for 3 h, and then processed as in B. Mitochondrial fractions were immunoblotted for PKM2 and Tom20 and blot images are shown. Images were quantified and expressed as fold change of control. n = 3 per group. F: WT macrophages were pretreated with 20 μg/ml oxLDL for 24 h or pretreated with 0.5 μM shikonin for 1 h, followed by addition of oxLDL and incubated for 24 h before mtROS assay (left panel) or phagocytosis assay (right panel). MFI was quantified and shown in the bar graph; n = 3–4 per group. ns, not significant; ∗*P* < 0.05; ∗∗*P* < 0.01; ∗∗∗*P* < 0.001; and ∗∗∗∗*P* < 0.0001. DAPI, 4′,6-diamidino-2-phenylindole; HMDM, human monocyte-derived macrophage; OxLDL, oxidized low-density lipoprotein; MFI, mean fluorescence intensity; mtROS, mitochondrial reactive oxygen species; PMK2, pyruvate kinase muscle 2.

The gene *PKM* encodes two different isoforms, PKM1 and PKM2, via RNA splicing (23). OxLDL selectively upregulated PKM2 but not PKM1 (supplemental Fig. S4A), prompting us to focus on PKM2 in subsequent mechanistic studies. While PKM2 is traditionally considered a cytosolic enzyme, oxLDL was previously reported to stimulate its nuclear translocation (24). Interestingly, we observed that oxLDL also promoted PKM2 colocalization with the mitochondrial marker Tom20 (Fig. 5A). While 51.4% of the mitochondria area displayed PKM2/Tom20 colocalization in control, oxLDL increased colocalization to 77.3%, suggesting more PKM2 translocated to the mitochondria.

To validate this finding, we performed mitochondrial and cytosolic fractionation experiments. As shown in supplemental Fig. S4B, the separation between the cytosolic and mitochondrial fractions was successfully achieved, with both fractions confirmed to be free from nuclear contamination. OxLDL nearly doubled mitochondrial PKM2 levels in HMDMs, without affecting cytosolic PKM2 levels (Fig. 5B). Time-course analysis in murine peritoneal macrophages showed that mitochondrial PKM2 levels increased within 1 h of oxLDL treatment, peaked at 3 h, and remained elevated for 24 h (supplemental Fig. S4C). Notably, oxLDL-stimulated PKM2 mitochondrial translocation was absent in *Cd36*-null macrophages (Fig. 5C), implicating CD36 in this process.

To test the role of PKM2 in mtROS induction, we used siRNA to knock down PKM2 in HMDMs (supplemental Fig. S4D). PKM2 knockdown significantly reduced oxLDL-induced mtROS and phagocytosis (Fig. 5D). Consistent with these findings, the PKM2 inhibitor shikonin (25) blocked oxLDL-induced mitochondrial translocation of PKM2 (Fig. 5E) but did not affect its nuclear translocation (supplemental Fig. S4E). Shikonin also inhibited oxLDL-induced mtROS and phagocytosis (Fig. 5F), supporting the role of PKM2 mitochondrial translocation in oxLDL-induced mtROS production and phagocytic activity.

### The chaperone protein GRP75 mediates PKM2 mitochondrial translocation

To investigate the molecular mechanism underlying PKM2 mitochondrial translocation, we performed mass spectrometry analysis on PKM2-interacting proteins. WT peritoneal macrophages were treated with 20 μg/ml oxLDL for 3 h, followed by IP of PKM2 using a specific antibody. SDS-PAGE–separated immunoprecipitates from control and oxLDL-treated samples were analyzed, identifying 26 mitochondrial proteins co-IP with PKM2 (supplemental Table S1).

**Fig. 6:**
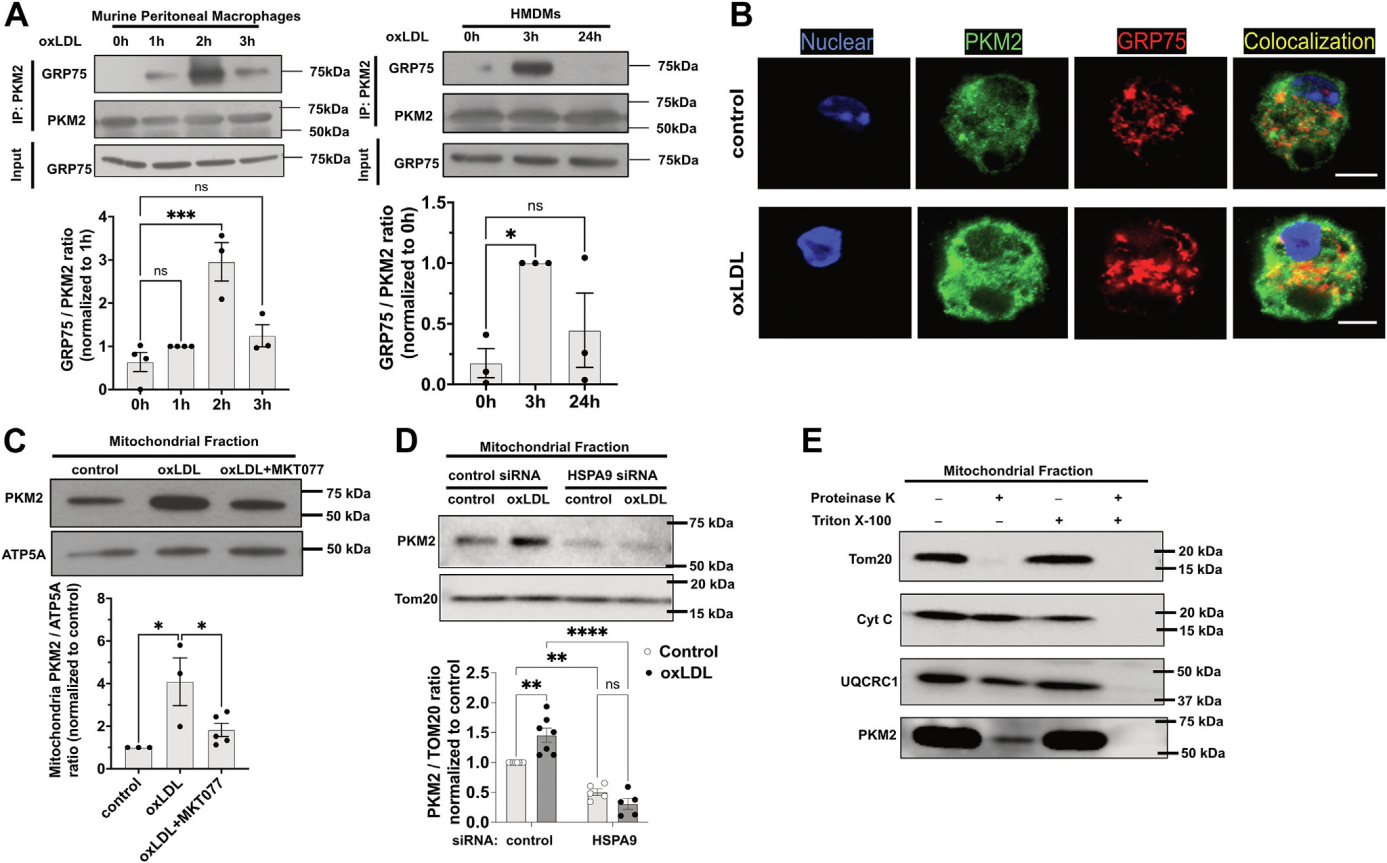
The chaperone protein GRP75 mediates PKM2 mitochondrial translocation. A: Murine peritoneal macrophages (left panel) or HMDMs (right panel) were treated with 20 μg/ml oxLDL or 50 μg/ml oxLDL, respectively, for indicated periods before cell lysates were IP with anti-PKM2. Representative Western blot images of GRP75 and PKM2 from PKM2 precipitates and GRP75 from total cell lysates (Input) are shown. Images were quantified and expressed as a fold change of 1 h. n = 3 per group. B: Representative confocal images of macrophages immunostained for PKM2 (green) and GRP75 (red). Nuclei were stained by DAPI (blue). Scale bar: 5 μm. C: WT macrophages were treated with 20 μg/ml oxLDL or pretreated with 1 μM MKT077, followed by addition of oxLDL for 3 h before cell fractionation. Mitochondrial fractions were immunoblotted for PKM2 and ATP5A and blot images are shown. Images were quantified and expressed as fold change of control. n = 3–5 per group. D: HMDMs transfected with control or *HSPA9* siRNA for 24 h were treated with 50 μg/ml oxLDL for additional 24 h. Mitochondrial fractions were subjected to immunoblot of PKM2 and Tom20. Images were quantified and expressed as fold change of control. n = 5–7 per group. E: OxLDL-pretreated WT macrophage mitochondria fractions were subjected to proteinase K digestion in the absence or presence of Triton X-100. Mitochondrial lysates were then subjected to immunoblot of Tom20, cytochrome C, UQCRC1, and PKM2. Representative blot images from four separate experiments were shown. n = 4 per group. ns, not significant; ∗*P* < 0.05; ∗∗*P* < 0.01; ∗∗∗*P* < 0.001; and ∗∗∗∗*P* < 0.0001. DAPI, 4′,6-diamidino-2-phenylindole; HMDM, human monocyte-derived macrophage; IP, immunoprecipitation; OxLDL, oxidized low-density lipoprotein; PMK2, pyruvate kinase muscle 2.

Among these proteins, GRP75, a chaperone of the HSP70 family known to facilitate protein import into mitochondria (26, 27), stood out. GRP75 levels in PKM2 IPs were 3-fold higher in oxLDL-treated samples than controls (supplemental Table S1). This enhanced PKM2/GRP75 interaction was confirmed in murine macrophages and HMDMs, with peak interaction observed 2–3 h post-oxLDL treatment (Fig. 6A). A control IgG antibody did not precipitate either PKM2 or GRP75, confirming the specificity of the interaction (supplemental Fig. S5A). Immunostaining further demonstrated increased colocalization of PKM2 and GRP75 following oxLDL exposure (Fig. 6B).

To test GRP75’s role, we used MKT-077, a selective GRP75 inhibitor of its chaperone function (28, 29). MKT-077 blocked oxLDL-induced PKM2 mitochondrial translocation (Fig. 6C) but did not affect nuclear localization of PKM2 (supplemental Fig. S4E). Similarly, GRP75 knockdown using *HSPA9* siRNA reduced GRP75 expression by 65% (supplemental Fig. S5B) and completely inhibited oxLDL-induced PKM2 mitochondrial translocation (Fig. 6D).

To determine whether PKM2 entered the mitochondria, we conducted a proteinase K digestion assay using a previously established protocol (30). Mitochondrial fractions from oxLDL-treated macrophages were digested by proteinase K before they were subjected to immunoblots of mitochondrial proteins and PKM2. A portion of mitochondrial PKM2, along with cytochrome C (intermembrane protein) and UQCRC1 (matrix protein), resisted digestion unless the mitochondrial membrane was disrupted with Triton X-100 (Fig. 6E). This suggests that some PKM2 proteins were protected within the mitochondrial membranes, confirming its intramitochondrial localization.

### Mitochondrial PKM2 binds to the electron transport chain complex III

**Fig. 7:**
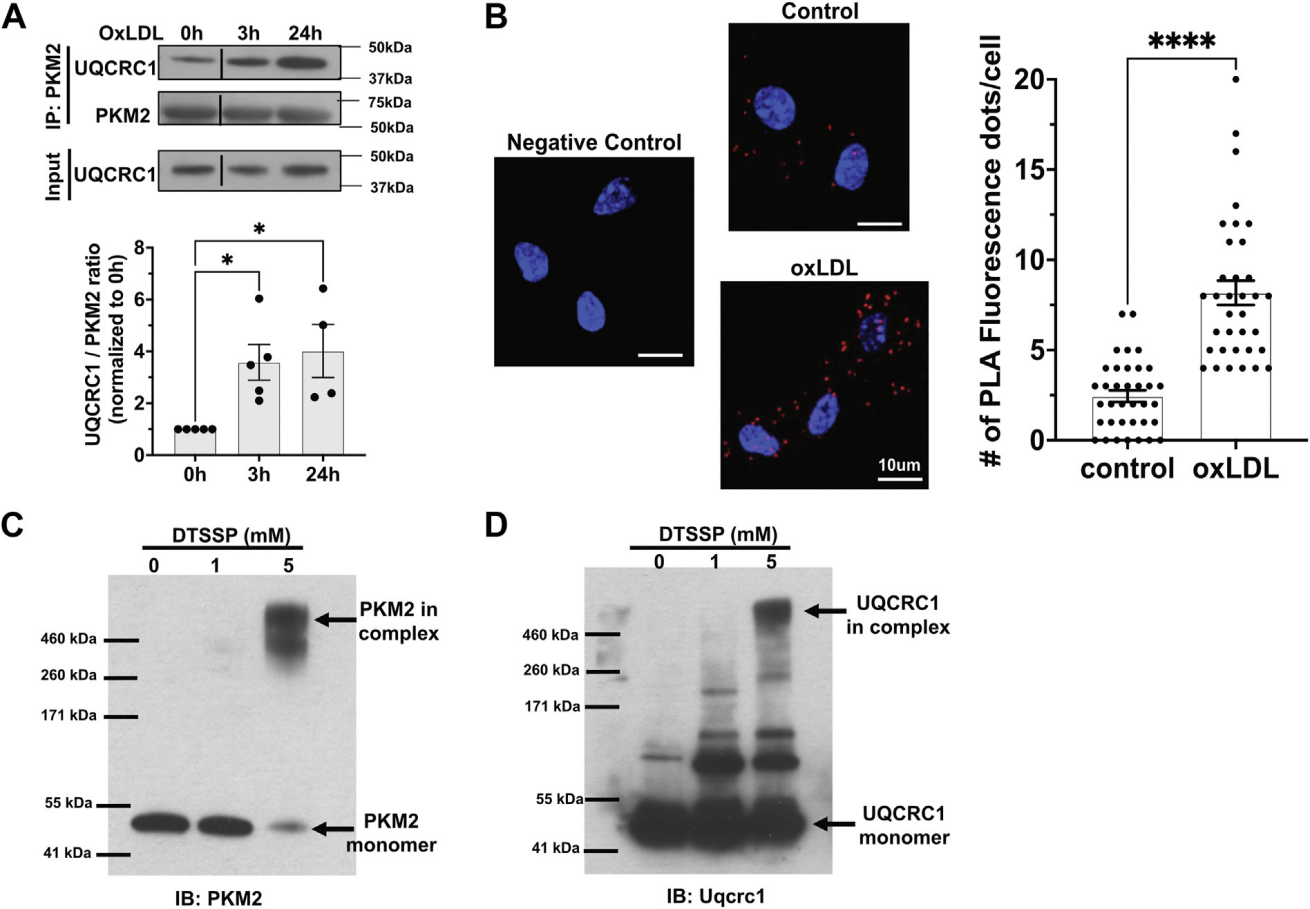
Mitochondrial PKM2 binds to the ETC complex III. A: WT peritoneal macrophages treated with 20 μg/ml oxLDL for indicated periods before cell lysates were IP with anti-PKM2. Representative Western blot images of UQCRC1 and PKM2 from PKM2 precipitates and UQCRC1 from total cell lysates (Input) are shown. Images were quantified and expressed as fold change of 0 h (control). n = 4–5 per group. B: Representative confocal images from in situ proximity ligation assay (PLA) show enhanced PKM2/UQCRC1 interaction with or without 20 μg/ml oxLDL 24 h treatment. The PLA signals (red fluorescence dots) were quantified and shown in the bar graph. n = 3. The PLA assay without primary antibody addition serves as a negative control. Scale bar: 10 μm. C: OxLDL-treated macrophage lysates were incubated with DTSSP crosslinker for 1 h at room temperature, followed by SDS-PAGE and immunoblot for PKM2. A representative blot image is shown. n = 5. D: The same treatment as in (C), followed by immunoblot for UQCRC1. A representative blot image is shown. n = 5. ns, not significant; ∗*P* < 0.05; and ∗∗∗∗, *P* < 0.0001. DTSSP, 3,3′-dithiobis(sulfosuccinimidyl propionate; ETC, electron transport chain; IP, immunoprecipitation; OxLDL, oxidized low-density lipoprotein; PMK2, pyruvate kinase muscle 2.

Electron transport chain (ETC) complex III is a major site of mtROS production (31). Our mass spectrometry analysis identified UQCRC1, a subunit of ETC complex III, exclusively in oxLDL-treated PKM2 immunoprecipitates (supplemental Table S1). To validate this interaction, PKM2 immunoprecipitates were analyzed by immunoblotting for UQCRC1, revealing increased UQCRC1 association with PKM2 starting at 3 h and peaking at 24 h after oxLDL treatment (Fig. 7A). An in situ proximity ligation assay corroborated these findings, showing enhanced PKM2/UQCRC1 interactions following oxLDL treatment (Fig. 7B).

To confirm a direct interaction between PKM2 and the whole complex, we employed a crosslinking approach using 3,3′-dithiobis(sulfosuccinimidyl propionate (DTSSP). In oxLDL-treated macrophage lysates, crosslinking with 5 mM DTSSP resulted in the disappearance of the PKM2 monomer band and the appearance of a high molecular weight band (Fig. 7C). This band corresponds to the combined molecular weight of ETC complex III (490 kDa) and PKM2 (58 kDa). Control experiments using purified PKM2 and DTSSP did not produce a high molecular weight band, ruling out PKM2 oligomer as this high molecular weight band (supplemental Fig. S5C). Additionally, UQCRC1 immunoblotting confirmed the presence of the same high molecular weight band in crosslinked samples (Fig. 7D).

These results indicate that mitochondrial PKM2 associates with ETC complex III, which may contribute to mtROS induction during oxLDL treatment, suggesting a potential mechanistic link.

## Discussion

In this study, we demonstrate that oxLDL promotes macrophage phagocytosis by inducing mtROS. We identify a novel molecular mechanism in which oxLDL/CD36 signaling drives the translocation of the cytosolic enzyme PKM2 to mitochondria, a process facilitated by the chaperone protein GRP75. Once localized to the mitochondria, PKM2 enhances mtROS production, thereby increasing phagocytic activity in response to oxLDL.

Atherosclerosis exemplifies a chronic inflammatory disease associated with hyperlipidemia, oxidative stress, and mitochondrial dysfunction (8). We previously demonstrated that oxLDL/CD36 signaling induces metabolic reprogramming from mitochondrial respiration to glycolysis, accompanied by mtROS elevation and proatherogenic phenotypes in macrophages (7). Additionally, we showed that mtROS levels are persistently elevated in circulating monocytes and aortic macrophages in a CD36-dependent manner, contributing to the initiation of atherosclerosis (7). In this study, we further revealed that aortic foamy macrophages (F4/80^+^Trem2^+^ subpopulation) exhibit both increased mtROS levels and enhanced phagocytic activity (Fig. 3). During phagocytosis, macrophages engulf pathogens, AC, cell debris, and other large extracellular bioparticles, which are subsequently degraded in lysosomes. The breakdown products, including lipids, are either metabolized or exported (32). However, when particle ingestion is excessive, as in atherogenic environments, the capacity of macrophages to process and efflux lipids becomes insufficient (33). This imbalance is further aggravated by CD36-mediated oxLDL endocytosis and overloading (5), resulting in the intracellular accumulation of lipids, foam cell formation, and chronic inflammation. Our findings suggest that mtROS may overstimulate phagocytosis in aortic macrophages, thereby contributing to atherogenesis. Therefore, strategies to limit mtROS production while preserving basal phagocytic activity may help restore the balance between macrophage lipid uptake and efflux, thereby reducing foam cell formation.

The exact identity of mtROS involved in oxLDL/CD36 signaling and phagocytosis regulation remains unresolved. Although MitoNeoD is considered selective for mitochondrial superoxide (13), the potential involvement of other oxidants cannot be excluded. Future studies utilizing LC-MS–based analysis to identify specific MitoNeoD oxidation products are necessary to clarify this question. Additionally, while MitoTEMPO is widely used as a mitochondrial superoxide scavenger, it may also interfere with peroxidation and scavenge other potent one-electron oxidants (34). Furthermore, the inhibitory effects observed with the MCAT transgenic strategy (Fig. 2C) suggest that hydrogen peroxide may play a role in the signaling events that facilitate phagocytosis.

Another unanswered question is how mtROS enhance particle ingestion. Using a novel label-free live-cell imaging technique, we observed that macrophages pretreated with oxLDL exhibited a highly dynamic and distorted phenotype during AC ingestion (supplemental Video S2, highlighted by red arrows), compared to the control cells (supplemental Video S1). These phenotypes may reflect altered actin cytoskeleton dynamics, as the rearrangement of actin filaments is a key driver of the phagocytic process (35). Notably, actin filaments can be reversibly modified by ROS, which plays a crucial role in actin regulation (36). Moreover, oxLDL/CD36 signaling has been reported to alter actin dynamics via ROS generation (14). Thus, we propose that the oxLDL/CD36 axis temporally and spatially regulates actin rearrangement through fine-tuning of mitochondrial fission/fusion events, mtROS induction, and the modification of actin or actin-associated motor proteins, which will be tested in future studies.

We demonstrate that PKM2, a rate-limiting glycolytic enzyme, translocates to the mitochondria and induces mtROS production (Fig. 5). These findings reveal PKM2 as a mediator of the cellular response to oxLDL, linking oxidative stress, metabolic reprogramming, and mitochondrial dysfunction in atherosclerosis. The proatherogenic role of PKM2 is supported by both clinical and experimental evidence: in patients with coronary artery disease, PKM2 is associated with increased mtROS production (37), and in mice, myeloid cell PKM2 contributes to atherogenesis (38). PKM2 subcellular localization is regulated by its oligomeric state and posttranslational modifications (39), suggesting that CD36 signaling may induce specific modifications that promote its mitochondrial translocation. Supporting this hypothesis, we show that shikonin, a PKM2 inhibitor, effectively blocks oxLDL-induced PKM2 mitochondrial translocation, mtROS production, and phagocytosis (Fig. 5). Shikonin’s known ability to reduce PKM2 phosphorylation and disrupt its dimerization and tetramerization (40, 41) may underlie this inhibition.

However, the exact mechanism that PKM2 induces mtROS remains to be uncovered. We show that mitochondrial PKM2 interacts with UQCRC1, a subunit of the ETC complex III (Fig. 7). This complex is a key part of the mitochondrial energy production system, and it normally transfers electrons in a controlled manner. However, if this process is disrupted, electrons can leak and react with oxygen to form superoxide, a type of ROS. There are several possible mechanisms by which PKM2 facilitates mtROS. PKM2 might enhance ROS production by altering the activity of complex III proteins, such as cytochrome b or the Rieske iron-sulfur protein, which play critical roles in electron transfer (42). If these proteins become imbalanced, unstable intermediates can accumulate and increase superoxide formation. PKM2 might also influence how efficiently mitochondria use energy, potentially leading to conditions that favor electron leakage. Alternatively, PKM2 may affect the availability of key molecules like ubiquinol, which complex III relies on to function properly. If ubiquinol levels or turnover are altered, it could lead to inefficient electron transfer and more ROS production. Further studies are needed to determine whether PKM2 directly affects complex III activity or acts through broader mitochondrial metabolic changes that indirectly increase mitochondrial ROS.

In summary, we identify a novel mechanism regulating mtROS and phagocytosis in proatherogenic macrophages, offering potential therapeutic strategies to restore lipid homeostasis and mitigate foam cell formation.

## Data availability

The authors declare that all supporting data and methods are available within the article and the Supplemental material. The scRNA-Seq data for reanalysis were retrieved from Gene Expression Omnibus under accession number GSE97310. The bulk RNA-Seq data for reanalysis were retrieved under the accession number GSE116239. Additional supporting data and methods are available from the corresponding authors upon reasonable request.

## Supplemental data

This article contains supplemental data.

## Conflict of interest

The authors declare that they have no conflict of interest with the contents of this article.
