# Supplementary material for: Oxidized LDL stimulates PKM2-mediated mtROS production and phagocytosis
Source: J Lipid Res. 2025 Apr 16;66(5):100809. doi: 10.1016/j.jlr.2025.100809 (PMC12142535; doi:10.1016/j.jlr.2025.100809)
Supplement: Supplemental Material [file mmc3.pdf]

SUPPLEMENTAL FIGURES

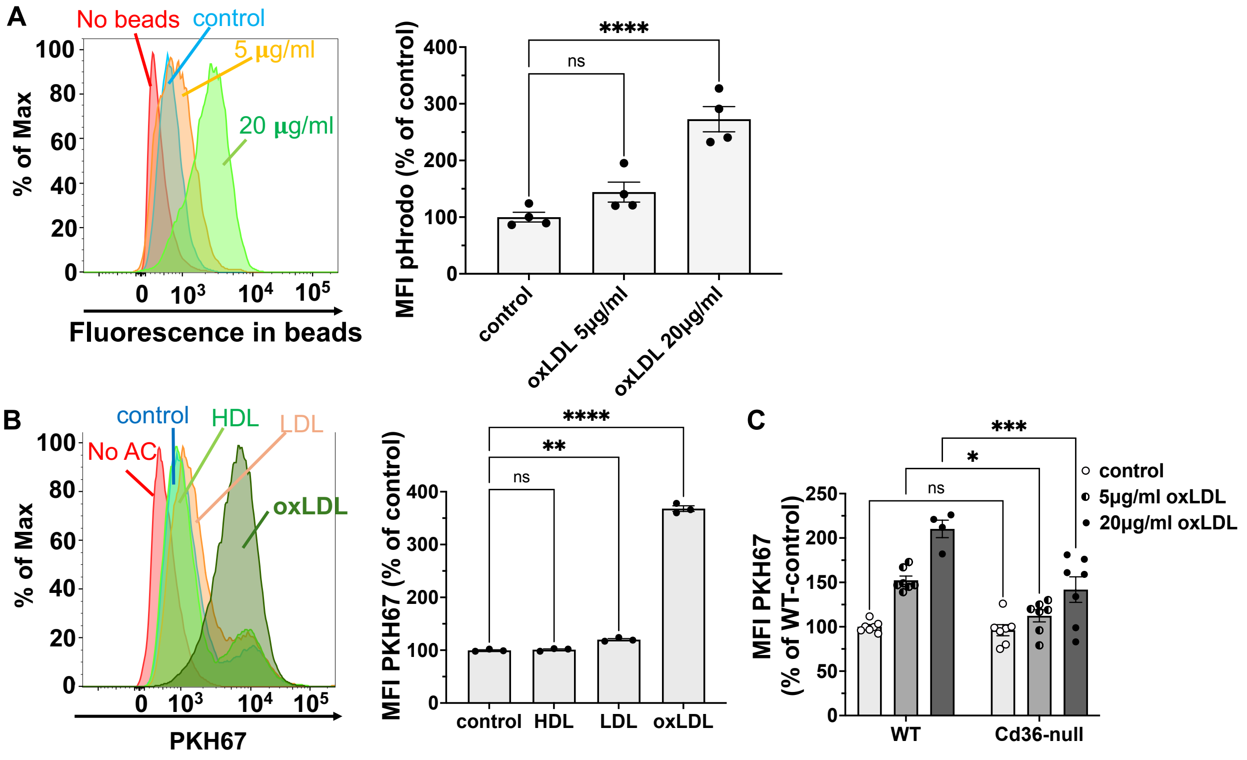

Figure S1. OxLDL stimulates phagocytosis in macrophages. (**A**) Examples of histograms of bead fluorescence in WT peritoneal macrophages pre-treated with 5 or 20 µg/ml oxLDL for 24h, followed by 10-µm green fluorescence beads co-incubation for 16 h (phagocytosis assay) and flow cytometry analysis. MFIs are shown in the bar graph; n=4 per group. (**B**) Examples of histograms of PKH67 fluorescence in WT peritoneal macrophages pre-treated with 20 µg/ml HDL or LDL or oxLDL for 24 h, followed by PKH67-labeled AC co-incubation for 15 min and flow cytometry analysis. The PKH67 MFI was quantified and shown in the bar graph; n=3 per group. (**C**) WT, or *Cd36*-null peritoneal macrophages were pre-treated with 5 or 20 µg/ml oxLDL for 24h before co-incubation with PKH67-labeled AC for 15 min as primary efferocytosis assay. The PKH67 MFI was quantified and shown in the bar graph; n=3-4 per group. Max, maximum fluorescence intensity. ns, not significant; *, $p<0.05$; **, $p<0.01$; ***, $p<0.001$; ****, $p<0.0001$.

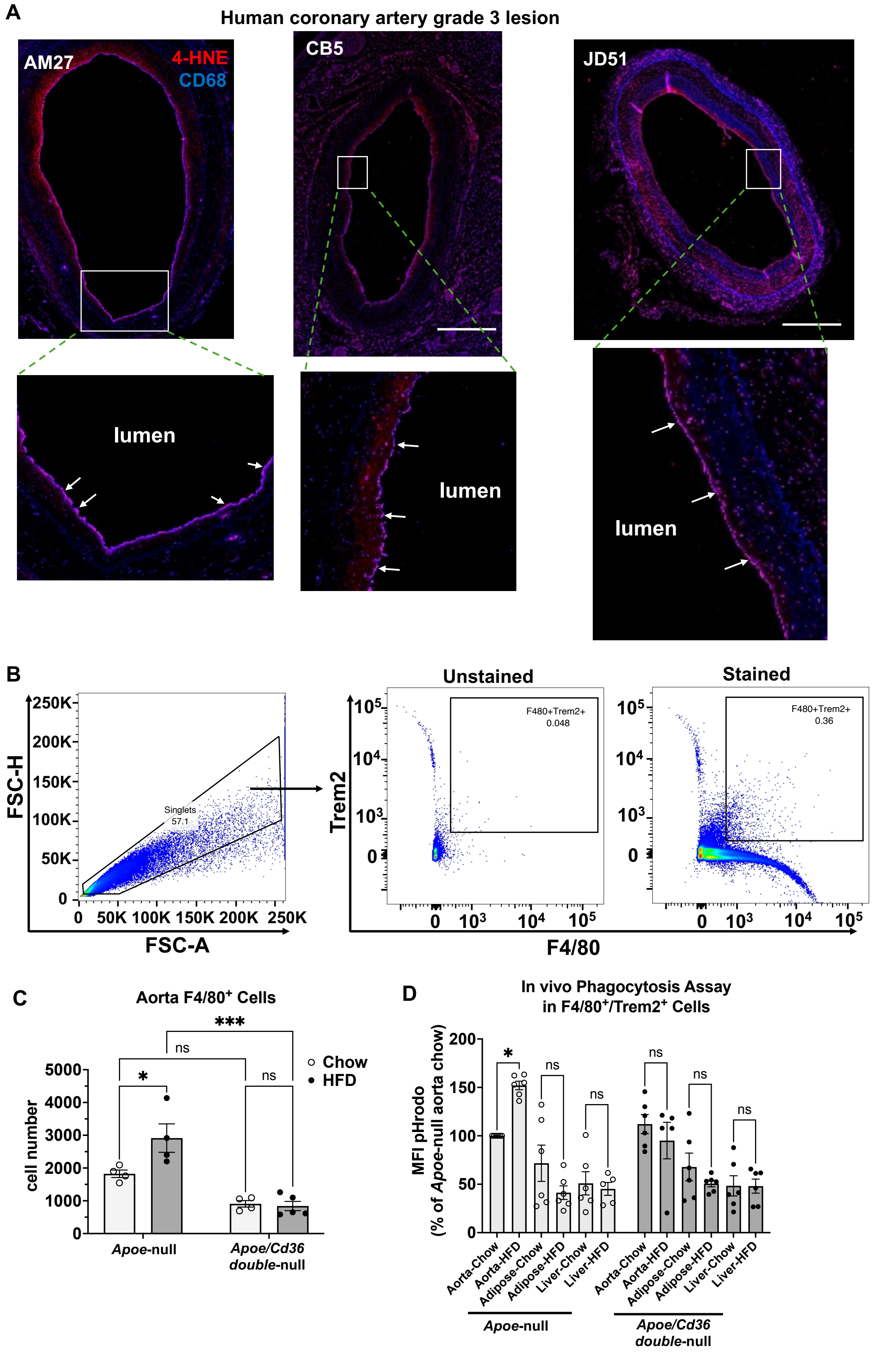

A
Human coronary artery grade 3 lesion
AM27
4-HNE
CD68
CB5
JD51
lumen
lumen
lumen
B
Singlets
57.1
FSC-H
FSC-A
Unstained
Stained
F480+Trem2+
0.048
F480+Trem2+
0.36
Trem2
F4/80
C
Aorta F4/80+ Cells
cell number
Chow
HFD
Apoe-null
Apoe/Cd36 double-null
D
In vivo Phagocytosis Assay in F4/80+/Trem2+ Cells
MFI pHrodo (% of Apoe-null aorta chow)
Aorta-Chow
Aorta-HFD
Adipose-Chow
Adipose-HFD
Liver-Chow
Liver-HFD
Apoe-null
Apoe/Cd36 double-null

Figure S2. Association of ROS signaling and phagocytosis in aortic macrophages. (**A**) Fluorescence images of human coronary arteries with grade 3 atherosclerosis lesions double stained with macrophage marker CD68 (blue) and oxidative stress marker 4-HNE (red). The white rectangle areas within the upper images were magnified and shown on the bottom. White arrows point to the colocalized positions in magenta color. AM27, CB5, and JD51 are ID labels of different individuals. Scale bar: 500 μm. (**B**) The gating strategy for selecting $F4/80^+$/$Trem2^+$ macrophages using flow cytometry. Cell clumps were excluded for further analysis using the FSC-A vs. FSC-H strategy. Then $F4/80^+$/$Trem2^+$ macrophages were identified based on a comparison of stained samples vs unstained samples. (**C**) Total amount of aortic $F4/80^+$ macrophages were quantified and shown in the bar graph; n=4-5 individual mice per group. (**D**), Aortas, adipose tissues, and livers are removed and digested into single-cell suspension for *in vivo* phagocytosis assay. Different tissue cells were then stained by anti-F4/80, anti-Trem2 antibodies. pHrodo MFI was quantified in different tissue-associated $F4/80^+$/$Trem2^+$ macrophages and shown in the bar graph; n=5-6 individual mice per group. ns, not significant; *, $p<0.05$; ***, $p<0.001$.

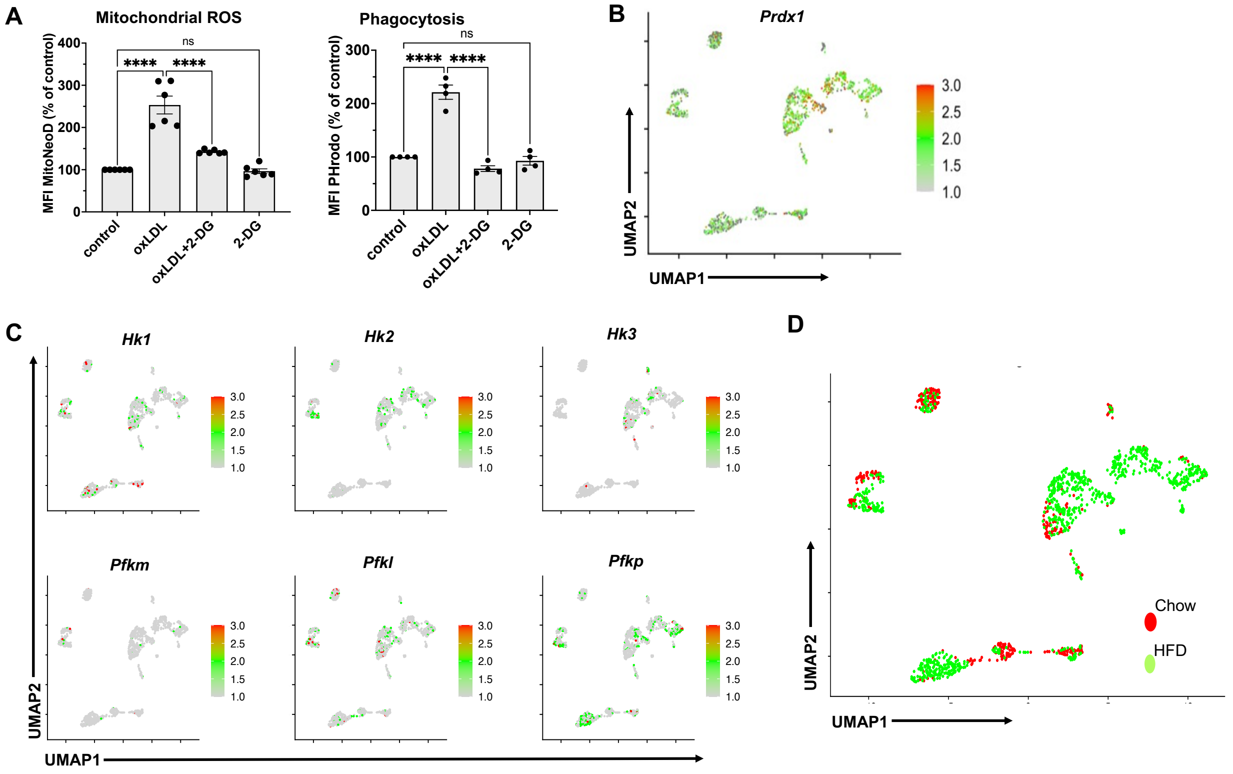

Figure S3. The connection of glycolysis with mtROS in immune cells. (**A**) WT peritoneal macrophages pre-treated with 20 µg/ml oxLDL or in combination with 10 mM 2-DG (1 h pre-treatment followed by co-incubation with oxLDL for 3 h) before mtROS or 24 h co-incubation before phagocytosis assay. The MitoNeoD (left) or pHrodo (right) MFI was quantified and shown in the bar graph; n=3 per group. (**B-C**) Mouse scRNA-seq data were re-analyzed as described in Figure 4. The expression pattern of the antioxidant gene *Prdx1* (B) and genes (*Hk1*, *Hk2*, *Hk3*, *Pfkm*, *Pfkl*, *Pfkp*) encoding two rate-limiting steps of glycolysis (C) is shown in the UMAP. (**D**) UMAP representation of cellular origins (chow diet vs HFD 11-week). ns, not significant; ****, p<0.0001.

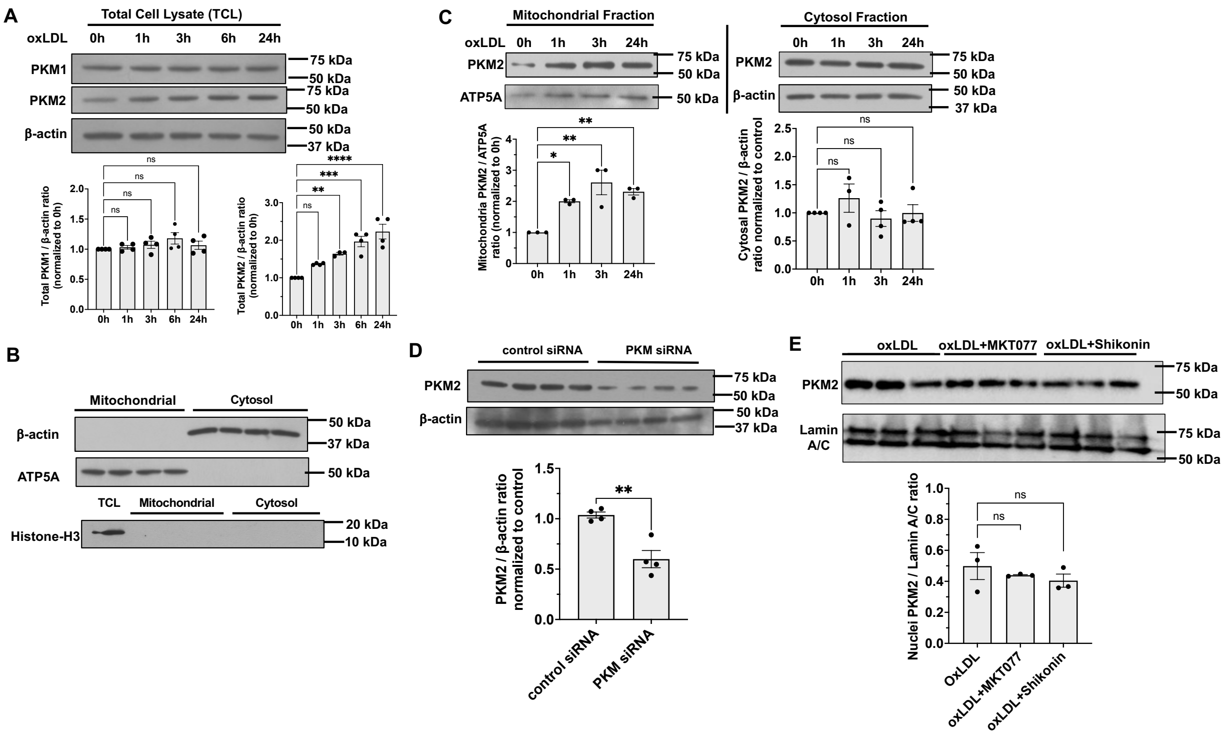

Figure S4. The glycolytic enzyme PKM2 translocates to the mitochondria. (**A**) WT macrophages were treated with 20 μg/ml oxLDL for indicated time periods. Representative Western blot images of PKM1, PKM2, and β-actin (loading control) were shown. Images were quantified and expressed as fold change of 0 h (control). n=4 per group. (**B**) Representative Western blot images of β-actin (cytosol marker), ATP5A (mitochondria marker) and Histone-H3 (nuclei marker) were shown from cell fractions. TCL: total cell lysate. (**C**) WT macrophages were treated with 20 μg/ml oxLDL for indicated time periods before cell fractionation. PKM2 and ATP5A (mitochondria fraction loading control) blot images from mitochondrial fractions were shown on the left. PKM2 and β-actin (cytosol fraction loading control) blot images from cytosol fractions were shown on the right. Images were quantified and expressed as fold change of control. n=3-4 per group. (**D**) HMDMs were transfected with PKM siRNA for 24 h before lysis. Representative Western blot images of PKM2 and β-actin were shown. Images were quantified and expressed as fold change of control. n=4 per group. (**E**) WT macrophages were treated with 20 μg/ml oxLDL or pre-treated with 1 μM MKT077 or 1 μM shikonin for 1 h followed by co-incubation with oxLDL for 3 h. Nuclei fractions of the cells were subjected to immunoblot of PKM2 and Lamin A/C (loading control). Images were quantified and shown in the bar graphs. ns, not significant; *, $p<0.05$; **, $p<0.01$; ***, $p<0.001$; ****, $p<0.0001$.

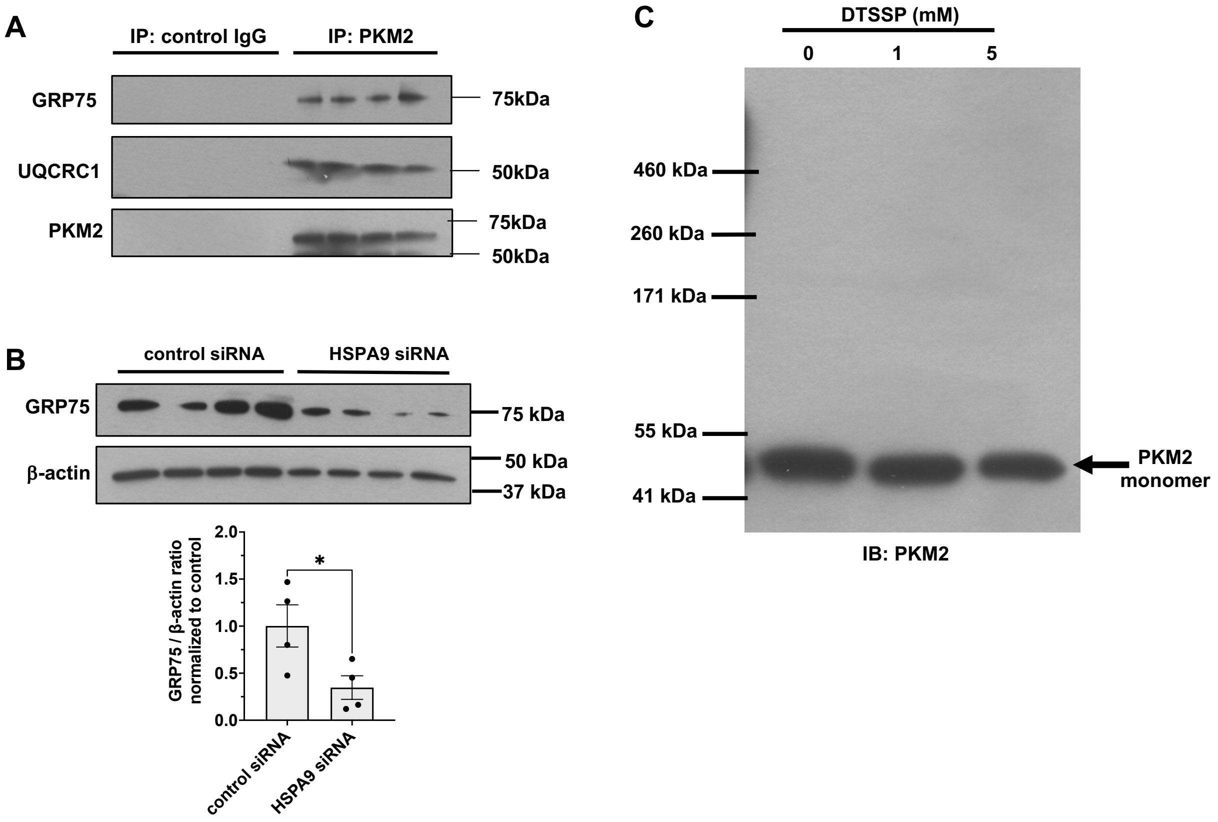

Figure S5. (**A**) Examples of Western blot images of GRP75, UQCRC1, and PKM2 from control IgG and anti-PKM2 IgG immunoprecipitates demonstrating specific bindings of PKM2 to GRP75 and UQCRC1. (**B**) HMDMs were transfected with HSPA9 siRNA for 24 h before lysis. Representative Western blot images of GRP75 and β-actin were shown. Images were quantified and expressed as fold change of control. n=4 per group. (**C**) Purified His-tagged human PKM2 proteins were incubated with DTSSP crosslinker for 1 h at room temperature, followed by SDS-PAGE and immunoblot for PKM2. A representative blot image is shown. n=5. *, $p<0.05$.

SUPPLEMENTAL TABLES

	Protein name	control intensity	oxLDL intensity
1	Slc25a12	0.0	5.0
2	Tufm	0.0	3.0
3	Uqcrc1	0.0	2.0
4	Ndufs3	0.0	1.0
5	Acadm	0.0	1.0
6	Acaa2	0.0	1.0
7	Cyc1	0.0	1.0
8	Letm1	0.0	1.0
9	Ndufs1	1.0	7.0
10	Slc25a13	1.0	5.0
11	Idh2	5.0	15.0
12	Grp75	2.0	6.0
13	Atp5c1	2.0	5.0
14	Decr1	2.0	5.0
15	Uqcrc2	2.0	4.0
16	Cpt1a	2.0	4.0
17	Sqor	8.0	13.0
18	Slc25a11	2.0	3.0
19	Slc25a1	2.0	3.0
20	Mdh2	19.0	23.0
21	Slc25a5	12.0	11.0
22	Hadhb	30.0	22.0
23	Hars2	7.0	5.0
24	Ndufv1	1.0	0.0
25	Nnt	1.0	0.0
26	Cyp11a1	1.0	0.0

Table S1. List of 26 mitochondrial proteins co-IP with PKM2. Two (UQCRC1 and GRP75, highlighted in red color) were picked for validation and further analysis.

Legends for Supplemental Videos

Video S1: This time-lapse video shows WT murine peritoneal macrophages (pointed by red arrows) interacting with apoptotic cell bodies (ACs, pointed by yellow arrows) in full culture medium (control). Images were captured every 15 seconds for 45 minutes using a NanoLive CX-A label-free Live Cell Imaging System and compiled into a 24-second video. This footage illustrates the normal behavior of macrophages during efferocytosis.

Video S2: This time-lapse video shows oxLDL-pretreated (20 μg/ml for 24h) WT murine peritoneal macrophages (pointed by red arrows) interacting with AC (pointed by yellow arrows). Images were captured using the same method as in Video S1 and compiled into a 23-second video. The macrophages exhibited a distorted morphology with thinner and longer filopodia. These phenotypes differ markedly from those of control macrophages (see Video S1). This footage illustrates that oxLDL alters the macrophage morphology.
